# Identification and Phylogenetic Characterization of Human Enteroviruses Isolated from Cases of Aseptic Meningitis in Brazil, 2013–2017

**DOI:** 10.3390/v11080690

**Published:** 2019-07-29

**Authors:** Emanuelle Ramalho, Ivanildo Sousa, Fernanda Burlandy, Eliane Costa, Amanda Dias, Roseane Serrano, Maria Oliveira, Renato Lopes, Maria Debur, Marion Burger, Irina Riediger, Maria L. Oliveira, Osvaldo Nascimento, Edson E. da Silva

**Affiliations:** 1 Laboratório de Enterovírus, Instituto Oswaldo Cruz, Fundação Oswaldo Cruz, Rio de Janeiro 21040900, Brazil; 2 Departamento de Neurologia, Faculdade de Medicina, Hospital Universitário Antônio Pedro, Universidade Federal Fluminense, Rio de Janeiro 24033900, Brazil; 3 Secretaria Estadual de Saúde de Pernambuco, Recife, PE 50751530, Brazil; 4 Setor de Virologia, Laboratório Central de Saúde Pública de Pernambuco, Recife, PE 50751530, Brazil; 5 Secretaria Estadual de Saúde do Paraná, Brazil—Divisão de Vigilância de doenças transmissíveis, Paraná 80230140, Brazil; 6 Laboratório Central de Saúde Pública do Estado do Paraná, Curitiba, Paraná 80060190, Brazil; 7 Centro de Epidemiologia da SMS, Curitiba, Paraná 80060130, Brazil; 8 Laboratory of Technological Development in Virology, Oswaldo Cruz, Institute, Fiocruz, Rio de Janeiro 21040900, Brazil

**Keywords:** aseptic meningitis, enterovirus, echovirus-30, echovirus-6

## Abstract

Aseptic meningitis is a common viral infection associated with human enteroviruses. The aim of the present study was to identify and characterize the enteroviruses associated with outbreaks and sporadic cases of aseptic meningitis that occurred in different regions of Brazil between 2013 and 2017. Cerebrospinal fluids obtained from patients admitted to public health facilities were analyzed. A total of 303 patients were positive for Human Enteroviruses (EV) by cell culture isolation with a median isolation rate throughout the year of 12%. We were able to identify enterovirus serotypes in 295 clinical specimens. Nineteen different serotypes were identified; the large majority corresponded to HEV-B species. Echovirus 30 (E-30) and Echovirus 6 (E-6) were the most prevalent genotypes (66.8%). Sequence analysis suggested that circulating E-30 was closely related to E-30 from other American countries; while E-6 was derived from Europe. Most of the patients consisted of children ≤ 15 years old. The temporal distribution of all aseptic meningitis and EV-positive cases showed an obvious seasonal pattern during autumn. Our results have provided valuable information about the enteroviral etiology of the aseptic meningitis cases in Brazil pointing to the importance of enterovirus surveillance in neurological diseases.

## 1. Introduction

Human enteroviruses (EV) are small non-enveloped RNA viruses belonging to the *picornaviridae* family and are classified into four species: EV-A, B, C, and D. Although most EV infections can be asymptomatic, they are associated with a broad spectrum of clinical presentations such as hand, foot, and mouth disease, acute myalgia, herpangina, conjunctivitis, upper and lower respiratory disease and severe syndromes of the central nervous system (CNS) [1,2]. Among the most common manifestations involving the CNS, it is worth mentioning encephalitis, acute flaccid paralysis, paralytic myelitis and aseptic meningitis [1].

Aseptic meningitis is defined as a non-bacterial acute infectious disease of the tissue that covers the brain and spinal cord and represents the most common viral infection of the CNS. The majority of aseptic meningitis cases are caused by non-polio enteroviruses, although other infectious and non-infectious causes can induce inflammation of the meninges [3]. The common symptoms in patients with viral meningitis can vary with age and underlying immune state, but include fever, irritability and lethargy.

In Brazil, a limited number of investigations on EV that are associated with aseptic meningitis outbreaks have been reported, such as those related to E-30 and E-13 [4,5]. These studies were based on a description of outbreaks, which have occurred in specific places within the country. In a specific geographical area, some EV serotypes may be endemic, causing recurrent epidemics. However, the study of the different geographical areas of a country with a large territory, as is the case for Brazil, may be necessary to understand the distribution of EV serotypes causing aseptic meningitis besides their clinical features. The present work aimed to identify and characterize the EV associated with aseptic meningitis outbreaks or sporadic cases in different geographic areas of Brazil between 2013 and 2017 with an emphasis on their epidemiological and genetic characteristics. The analyses were performed in the context of nationwide surveillance of EV.

## 2. Materials and Methods

### 2.1. Ethics Statement

The Enterovirus laboratory at FIOCRUZ is an official Brazilian Ministry of Health Reference Laboratory. The virus isolates utilized in the present study were part of the virus collection of the laboratory. Patients were attended to by the local public health system ambulatories where the clinical samples were collected. This activity was considered to be a public health response to EV surveillance and thus did not require a review by the review board.

### 2.2. Study Background

A retrospective study was conducted based on data collected by the national surveillance system for notifiable diseases and the laboratory database (patient’s demographical data, clinical symptoms) from 2013 to 2017. All cases reported to the surveillance system and all laboratorial data obtained from the analysis of 2659 aseptic meningitis suspected cases during this period were included in the study. A case was defined as meningitis-suspected based upon the presence of the following symptoms: Headache, fever, neck stiffness and vomiting, sometimes accompanied by nausea, cough, abdominal pain and weakness. Cerebrospinal fluid (CSF) was collected from individuals admitted to the public health system and sent to our laboratory for diagnosis. In general, the CSF specimens were maintained at about −20 °C during sample transport and stored at −70 °C at the laboratory. Clinical specimens were obtained from three out of the five geographic regions of the country (Northeast, Southeast and Southern).

### 2.3. Virus Isolation and Molecular Typing

95% of the CSF specimens (2532/2659) with sufficient volume were used to inoculate human rhabdomyosarcoma (RD) and human cervix carcinoma (Hep2C) cell lines as previously described (6). Cells were incubated at 37 °C and examined daily regarding the presence of cytopathic effect (CPE). After 7-days (if necessary), the cell cultures were frozen, thawed and re-inoculated and another 7-days examination was performed. Culture supernatants showing CPE were collected and frozen at −70 °C. EV-positive was defined as CPE-positive followed by conventional reverse transcriptase polymerase chain reaction (RT-PCR) for EV.

To identify the EV serotypes associated with aseptic meningitis, viral RNA was extracted from 140 μL of the cell culture EV-positive using the QIAamp viral RNA mini kit (Qiagen, Hilden, Germany) and used as a template for cDNA construction using random primers (Promega, Madison, WI, USA) and SuperScript III Reverse Transcriptase (Invitrogen, Calrsbad, CA, USA) according to the manufacturer’s instructions. RT-PCR was performed using the primer pairs 224-011 and 008-011 (for entire VP1) in order to amplify the VP1 gene [4]. After 32 cycles (94 °C for 30 s, 42 °C for 30 s, and 60 °C for 2 min) in a model 9700 thermocycler (Applied Biosystems, Foster City, CA, USA), the PCR products were subjected to electrophoresis in 1% agarose to identify samples with positive amplicons. Specific products were gel-purified using the QIAquick Gel Extraction Kit (QIAGEN) and quantified with the Low DNA Mass Ladder (Invitrogen) in a 1% agarose gel. Cycle sequencing reactions were performed by using the ABI PRISM BigDye Terminator v3.1 Cycle Sequencing Ready Reaction Kit (Applied Biosystems) in a GeneAmp PCR System 9700 thermocycler (Applied Biosystems) with 25 cycles of 96 °C for 25 s, 42 °C for 30 s, and 60 °C for 3 min. Products were precipitated and analyzed as previously described [6]. To determine the EV serotype, the partial or complete VP1 sequences of the enterovirus isolates were compared with sequences available in GenBank.

### 2.4. Phylogenetic Analysis

Brazilian VP1 sequences from Echovirus 6 (N = 63, 867pb) and Echovirus 30 (N = 80, 876 bp) were aligned with reference and representative sequences using Muscle [7], available in the Mega 7.0 package [8]. Reference and representative sequences were blasted, downloaded from Genbank (https://www.ncbi.nlm.nih.gov/nucleotide/) and added to our datasets (the accession numbers are described in the respective phylogenetic trees). Furthermore, a North-American CVA6 sequence (AF081297) was used as an outgroup in the Maximum Likelihood (ML) tree. For each dataset, the best nucleotide substitution model was determined by JModeltest, version 2.1.4 [9,10]. For viral genotyping, the phylogenetic tree was reconstructed using a Maximum Likelihood algorithm (PhyML, v 3.0) [9,11], with a GTR+G+I nucleotide substitution model and an aLRT SH-like, as the fast likelihood-based method [12]. Temporal phylogenetic trees were reconstructed by a Bayesian Markov Chain Monte Carlo (MCMC) method, accessible in the BEAST software package, version 1.8.4 [13,14]. Time calibration was set based on the year of sample collection – available for all sequences, and the general time reversible (GTR) with gamma-distributed rates and invariant sites were employed as the nucleotide substitution model. Beast runs were carried out using the uncorrelated lognormal relaxed molecular clock model and a time-aware Gaussian Markov Random Field (GMRF) Bayesian skyride coalescent tree prior [15,16]. The length of MCMC chains were established as 30 and 40 million, sampled every 30,000 and 40,000 steps for Echovirus 6 and Echovirus 30, respectively. Trace files generated through Bayesian phylogenetic inference were visualized and analyzed in Tracer version 1.7 [17]. Convergence of parameters was considered in the presence of effective sample size (ESS) values exceeding 200. The target Maximum Clade Credibility (MCC) tree was summarized by TreeAnnotator 1.8.4, with a burn-in corresponding to 10% of states. MCC trees were visualized and edited in FigTree, version 1.4.3 Nucleotide sequences were deposited in GenBank with the accession numbers: MK570308-MK570388 (E-30) and MK570389-MK570451 (E-6).

## 3. Results

### 3.1. Epidemiological Analysis

A total of 2659 cases of aseptic meningitis were reported from 2013 to 2017 in four geographic regions of the country. Among these cases, 58.4% were from males with minimum and maximum values between 56.4% (2013) and 59.4% (2016), respectively. Among confirmed cases (EV-positive) from 2013 to 2017, the median patient age was five (range = 1–12 years), six (range = 1 month–57 years), four (range = 1month–38 years), four (range = 1 month–54 years) and four (range = 0–33 years) years, respectively.

**Figure 1:**
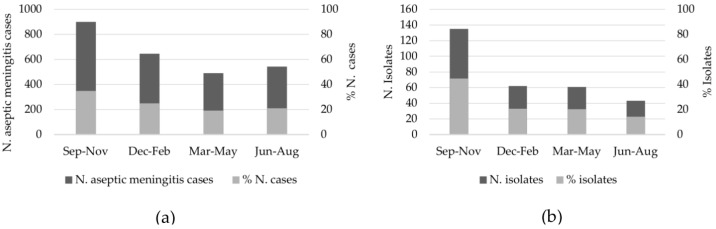
Seasonal distribution of aseptic meningitis cases in Brazil from 2013 to 2017 for all patients (**a**) and EV-positive patients (**b**).

The gender ratio (male/female) was 1.4:1, indicating a higher prevalence of aseptic meningitis in male children (Table 1). Regarding the EV-positive patients, a similar gender ratio was observed (1.5:1; ranged from 1.2 to 2.2) (Table 1). In general, the age distribution of aseptic meningitis cases ranged from 1 month to 92 years old (all patients) and 1 month to 38 years old (EV-positive patients). Most patients (69.2%, 1841/2659 for all patients and 94.7%, 287/303 for EV-positive patients) were children ≤15 years old (Table 1). The South region represented more than 81% that the specimens sent for enterovirus investigation. The monthly distributions of aseptic meningitis cases are illustrated in Figure 1. Although the number of cases was distributed throughout the year, we verified an increase in the reported cases during the spring months, which corresponded to 35% (900/2580) of all cases with the onset and specimen collection reported. (Figure 1a). The same profile was observed with samples from EV-positive patients, which showed a seasonal variation that was more prominent (45%, 135/301) during the spring (September–November) months (Figure 1b).

### 3.2. Enterovirus Identification

**Figure 2:**
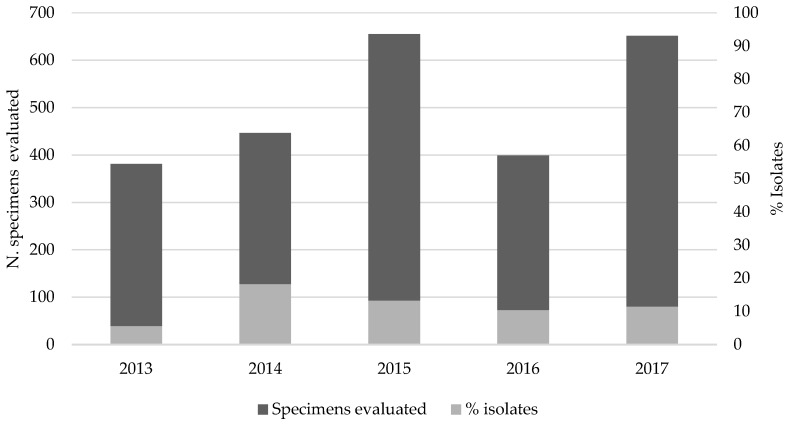
Proportion of enterovirus detection based on cell culture isolation in aseptic meningitis patients.

**Figure 3:**
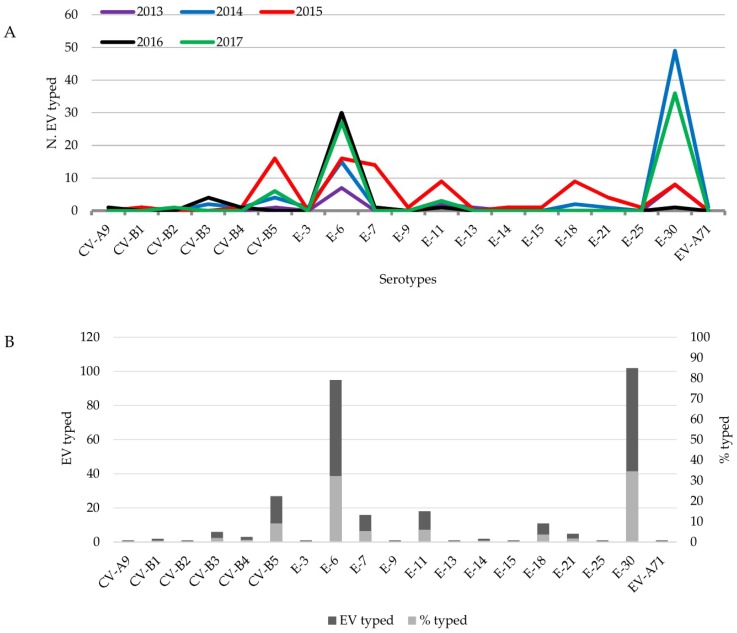
Enterovirus serotypes detected between 2013 and 2017 from aseptic meningitis patients. (**A**). Enterovirus serotypes identified per year (2013–2017). (**B**). Total number of typed enterovirus and relative number per serotype.

During the last five years (2013–2017), 303 EV isolates were detected based on virus isolation with an EV-positive rate ranging from 5.5% in 2013 (21/381) to 18.2% in 2014 (81/446) and a median throughout of the year of 12% (303/2532) (Figure 2). VP1 amplification was performed to identify the serotypes involved and revealed 19 different EV serotypes (Figure 3). Of these, 99.3% (293/295) were EV-B: CV-B1 (2), CV-B2 (1), CV-B3 (6), CV-B4 (3); CV-B5 (27); E-3 (1), E-6 (95), E-7 (16), E-9 (1), E-11 (18), E-13 (1), E-14 (2), E-15 (1), E-18 (11), E-21 (5), E-25 (1), E-30 (102). CV-A9 (1) and EV-71 (1), both belonged to EV-A, which were also detected (Figure 3A). The other eight specimens could not be identified due to failure to produce amplicons and were therefore considered untypable. In addition, 71 specimens that generated CPE in cell culture were classified as non-enterovirus based on PCR results.

E-30 (102/295; 34.6%) and E-6 (95/295; 32.2%) were the most frequently detected serotype from the EV-positive patients (Figure 3B). In spite of the higher incidence of the E-6 and E-30 serotypes during the years 2013 (15/20; 75%), 2014 (64/81; 79%), 2016 (31/39; 79.5%) and 2017 (63/73; 86.3%) (Figure 3A), 2015 did not show a high prevalence for these serotypes. Instead, we verified a more regular distribution throughout of the year of many serotypes, such as CV-B5, E-6, E-7, E-11, E-18 and E-30 (Figure 3A).

### 3.3. Phylogenetic Analysis

**Figure 4:**
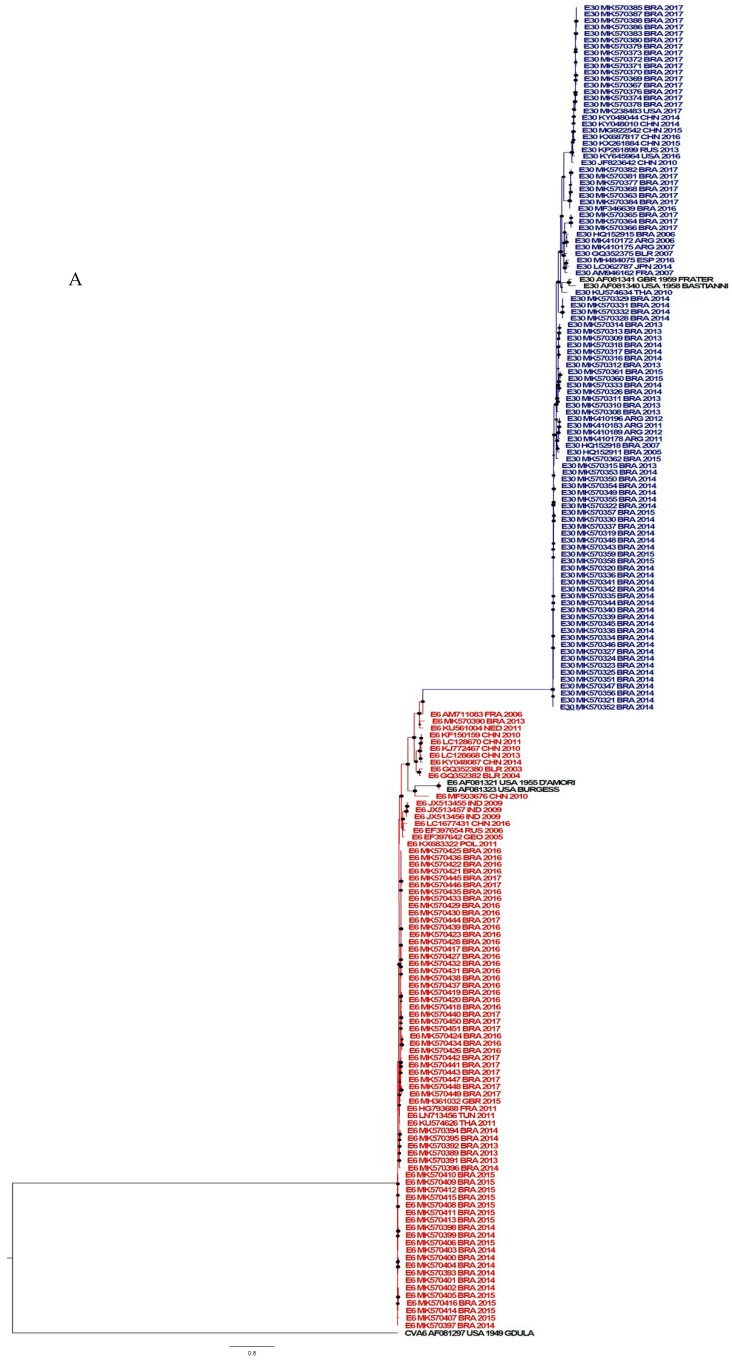

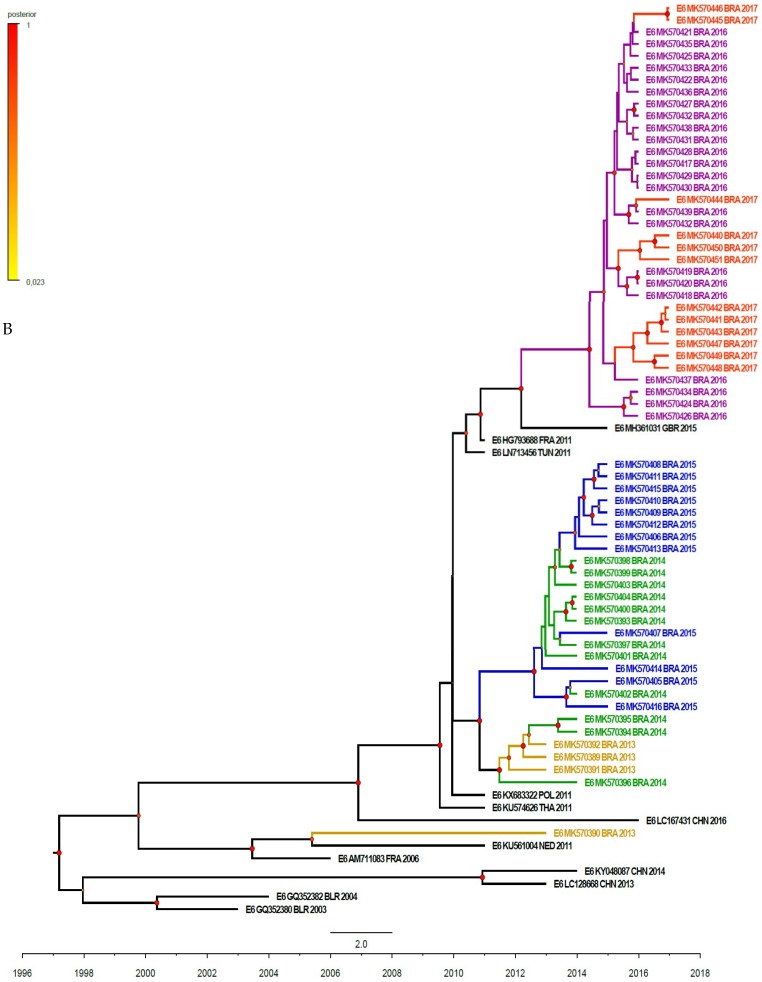

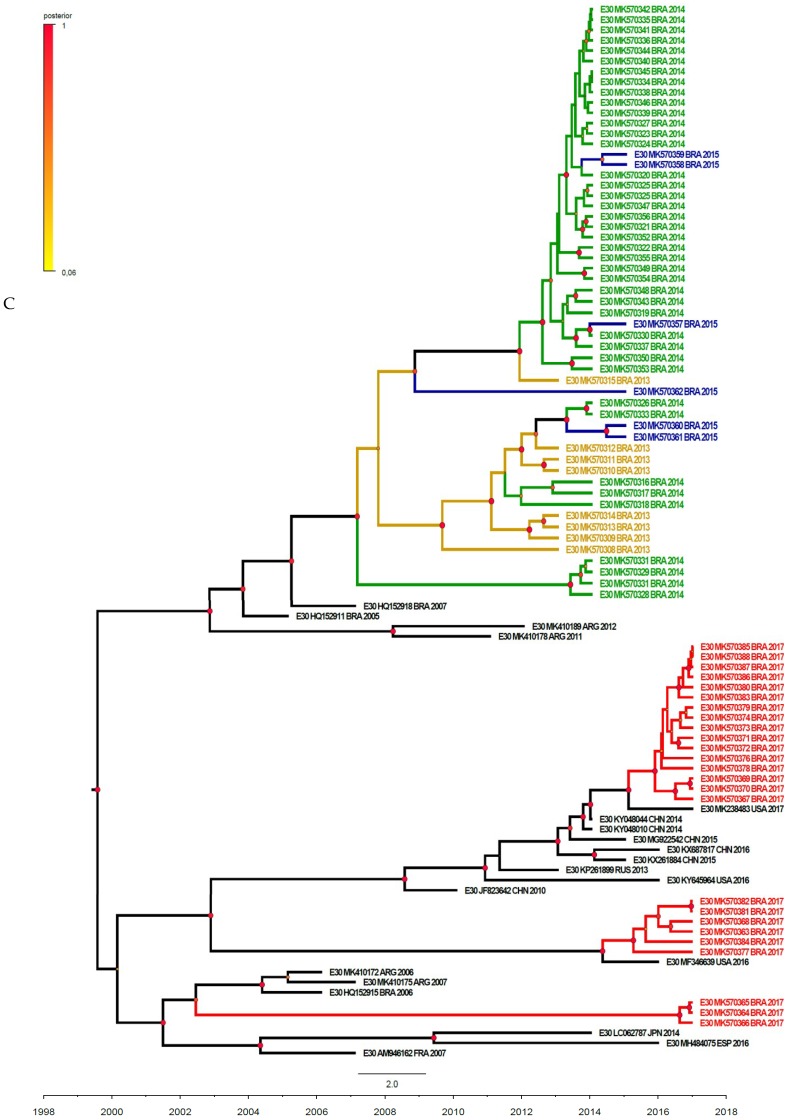
Phylogenetic analysis based on E-30 and E-6 VP1 sequences (876 bp and 867 bp in length, respectively) of Brazilian isolates causing meningitis and other sequences available at the GenBank database. (**A**) Phylogenetic analysis by maximum likelihood method of VP1 complete sequences. The B species is represented by the blue (E-30) and red (E-6) color. A species (outgroup) and prototypes are represented by black color. (**B**,**C**) Maximum clade credibility (MCC) tree based on the Bayesian analysis of the VP1 nucleotide sequences and their closely related sequences. The isolates of the same year are represented by a similar color. Branches are in time scale (year). Posterior probabilities are shown as a color scale.

The phylogenetic analysis was performed and the strains evaluated in this study were classified as shown in Figure 4A. The reconstructed phylogenetic tree from E-6 viruses (VP1 gene) circulating in Brazil from 2013 to 2017 is shown in Figure 4B. The Brazilian sequences were grouped into three main clusters and were genetically related to European viruses. In the first and minor clade, the Brazilian MK570390/2013 grouped with an isolate from Nederland (2011), both shared a common ancestor in 2005. The second cluster was mainly composed of Brazilian viruses circulating from 2013 to 2015. In 2010, a divergence event originated two subgroups: One that circulated in 2013 and another in 2014 to 2015, with the time of the most recent common ancestor (tMRCA) estimated in 2011 and 2012, respectively. The last cluster (2016–2017) showed a clear phylogenetic relationship with MH361032/2015, an isolate from Great Britain, with the year of divergence estimated as 2014. The co-circulation of viruses belonging to distinct clades was solely observed in 2013. Afterwards, the genetic clade 2014–2015 was replaced by the latest (2016–2017). Our data has proposed an important European role in the introduction of E-6 in Brazil, through multiple events.

Figure 4C presents the phylogenetic relationships of E-30 isolated in Brazil, based on the VP1 gene sequences. Two main genetic clades were identified: The first composed of viruses circulating from 2013 to 2015 and the other, included Brazilian isolates from 2017. Within each clade, subclades could be identified within the country, suggesting a higher genetic diversity, when compared to E-6. The first clade shared a common ancestor with Argentinian viruses, dated as 2002. Afterwards, circulation of this viral population was detected earlier in Brazil (2005) and later in Argentina (2011/2012). From 2007, virological surveillance allowed the identification of three subclades. Two out of three included isolates from 2013 to 2015 (BRA/2013/MK570308 and BRA/2013/570315, tMRCA in 2009 and 2011, respectively). Therefore, co-circulation of both variants could be observed in Brazil. The third circulated only in 2014 (BRA/2014/MK570328), with a tMRCA estimated in 2013. The second clade was composed of three subclusters, which diverged in 1999 and co-circulated during 2017. Two out of three were genetically related to North American viruses (USA/2016-MF346639 and USA/2017-MK238243), with tMRCA corresponding to 2015 and 2014, respectively. The third 2017 Brazilian clade was related to viruses previously identified in Argentina and Brazil in 2006 to 2007, with the year of divergence estimated as 2002. Altogether, these findings have suggested that distinct introduction events occurred in 2017, and also highlighting the protagonist of the American countries in this context.

Additionally, we evaluated the identities of E-30 and E-6 isolates with their prototype strains. As suggested, the cutoff value for unambiguous typing was 75% and 88% for nucleotide and amino acids identities, respectively. In this work, E-30 isolates revealed 80.4% to 83.2% and 91% to 93 % VP1 nucleotide (876nt) and amino acids (292 aa) identities and similarity, respectively. E6 isolates revealed 76.3% to 78.3% and 91% to 94.1% nucleotide and amino acids identities and similarity, respectively.

## 4. Discussion

Numerous studies about aseptic meningitis have been conducted over the last few years and non-polio enteroviruses have mostly been reported as an important pathogen causing many outbreaks [3,4,18,19]. In this study, we evaluated the epidemiological and virological features of aseptic meningitis outbreaks/sporadic cases caused by EV from 2013 to 2017 in Brazil. Enteroviruses were isolated from 12% (303/2532) of the patients and the EV-B species were the most common EV among the pathogens identified (99.3%; 293/295), while no EV-C species were detected. More than 90% (283/303) of the EV-positive patients in this study were under fifteen years of age. Our results were very similar to studies performed in other countries [19,20]. A predominance of aseptic meningitis among males was observed in this work with an M/F ratio of approximately 1.5, which was also consistent with the findings of other researchers [19,21]. In addition, as reported previously, we also observed a high incidence of EV-positive cases in patients with a median age of six years old [21,22,23]. Regarding the distribution of aseptic meningitis cases reported in different geographic areas of Brazil, we observed that the South region was responsible for more than 80% (2174/2659) of these cases. However, although aseptic meningitis is a notifiable disease in Brazil many regions do not refer CSF specimens to EV investigation, potentially underestimating the number of cases per region. 

Regarding the seasonal distribution of the aseptic meningitis cases, some studies in temperate climates have shown that enterovirus infections are observed during the summer and autumn seasons, whereas in tropical areas, the infections occur throughout the year [24]. In this study, we observed that 45% (135/301) of the EV-positive cases occurred from September to November (spring season), although cases also occurred in other seasons. Conversely, other studies reported that aseptic meningitis cases in the southern hemisphere countries occurred more frequently during the summer season, such as Australia [25] and South Africa [26]. The discrepancy in this seasonal distribution could be due to whether the source of specimen is from a sporadic case or from an outbreak.

According to previous studies, E-30 and E-6 were considered the major enteroviruses causing epidemics/sporadic cases of aseptic meningitis around of the world as well as a high prevalence of CV-B5 [19,20,21,22,27]. In the present work, we found that E-30 was the main pathogen associated with aseptic meningitis (102/295; 34.6% of the EV-positive patients) followed by E-6 (95/295; 32.2% of the EV-positive patients) suggesting a high occurrence of these serotypes during 2014 to 2017. As shown, these two enteroviruses were commonly observed in co-circulation in aseptic meningitis cases, enabling the occurrence of the co-infection, which may have favored recombination events between both of the strains. In fact, a prior study demonstrated recombination events between E-30 to E-6 lineages in a co-circulation scenario [28]. These events could be associated to different clinical features or viral tropism during the infection. Recently, E-30 was associated with an outbreak of acute myalgia and rhabdomyolisis, a condition not yet related to this serotype [2].

In the specific case of Brazil, E-30 was first described to be involved with aseptic meningitis outbreaks in Southern Brazil between 1998 and 2003 [27]. Subsequently, other outbreaks occurred due to E-30 in different geographic areas of the country, such as the Southeast region (Rio de Janeiro state), and the Northeast region (Pernambuco state) [4,29].

In this work, the phylogenetic analysis revealed that E-30 strains were grouped mainly into four clusters, where clusters 1 and 2 showed a close relationship with each other and with previously reported Brazilian sequences [4], whereas the other clusters segregated with E-30 strains from Argentina and USA.

Information about E-6-causing aseptic meningitis in Brazil is scarce. Dos Santos et al. reported a low incidence of E-6 in aseptic meningitis outbreaks/sporadic cases in Brazil between 1998 and 2003 [27]. On the other hand, a previous study demonstrated the involvement of E-6 in an aseptic meningitis outbreak that occurred in the São Paulo state in 2004 [30]. In fact, we identified the increasing occurrence of E-6 in aseptic meningitis cases in Brazil. In the last years, the number of E-6 cases detected raised from 7 (2013) to 30 (2016), representing an almost 5-fold increase in the detection rate. Furthermore, E-6 was the main EV involved in aseptic meningitis cases identified across 2015 and 2016. Phylogenetic analysis revealed that E-6 converged in two highly related clusters with a close genetic relationship to each other and with E-6 strains identified from European patients. Our results have suggested that the Brazilian E-6 viruses were derivatives from the ones previously circulating in Europe. 

The typing of non-polio enteroviruses is conventionally dependent on virus isolation in cell culture. However, the major drawback is that enteroviruses are frequently not identified due to the fact that some of them replicate poorly in culture [31]. Nowadays, cell culture isolation has been supplanted by qRT-PCR for routine detection of enterovirus infection. As related previously, we also observed that many clinical samples were negative in cell culture revealing a very low efficiency of isolation [32,33]. However, other factors could explain the lower efficiency in the enterovirus isolation rate, such as prolonged storage of clinical specimens and delays in their transportation to the laboratory. In this study, we had 71 samples that generated CPE in the cell culture but had EV-negative results in the PCR assay. Other viruses, such as human herpes viruses (HHV 1-5) and arboviruses might be associated with these cases. Other studies are currently in progress in our laboratory to identify the causative agents.

Overall, the enteroviral etiology and epidemiologic characteristics involved in aseptic meningitis in Brazil is similar to that observed around the world. E-30 and E-6 are predominant serotypes involved in aseptic meningitis cases. The Brazilian vast territory associated with recurrent E-6/E-30 detection throughout the years, and the high recombination capacity of these viruses showed the importance of enterovirus surveillance in the aseptic meningitis context.

## Figures and Tables

**Table 1:** Demographic features of aseptic meningitis patients in Brazil, 2013–2017.

Variable	Year (All Patients/EV-Positive)				
Year	2013	2014	2015	2016	2017
**Year**					
**≤15 year**	287/21	318/73	530/84	247/39	459/70
**Region**					
**South**	358/14 (3.9) *	365/67 (18.3)	464/56 (12)	399/38 (9.5)	588/61 (10.4)
**Southeast**	3/0 (0)	13/6 (46.1)	3/0 (0)	5/0 (0)	24/2 (8.3)
**Northeast**	45/7 (15.5)	79/8 (10.1)	229/30 (13.1)	5/3 (60)	50/11 (22)
**Midwest**	2/0 (0)	8/0 (0)	11/0 (0)	7/0 (0)	1/0 (0)
**Sex**					
**Male**	230/12 (5.2)	275/47 (17)	420/59 (14)	247/23 (9.3)	380/40 (10.5)
**Female**	178/9 (5)	190/34 (17.9)	287/27 (9.4)	169/18 (10.6)	283/34 (12)
**Total**	408/21	465/81	707/86	416/41	663/74

* % positive.
